# Supplementary material for: Spatial-temporal dynamics of a microbial cooperative behavior resistant to cheating
Source: Nat Commun. 2022 Feb 7;13:721. doi: 10.1038/s41467-022-28321-9 (PMC8821651; doi:10.1038/s41467-022-28321-9)
Supplement: Supplementary file 1 — Supplementary Information [file 41467_2022_28321_MOESM1_ESM.pdf]

Supplementary Information for

"Spatial-temporal dynamics of a microbial cooperative behavior resistant to cheating"

By: Hilary Monaco, Kevin S. Liu, Tiago Sereno, Maxime Deforet,
Bradford P. Taylor, Yanyan Chen, Caleb C. Reagor, Joao B. Xavier

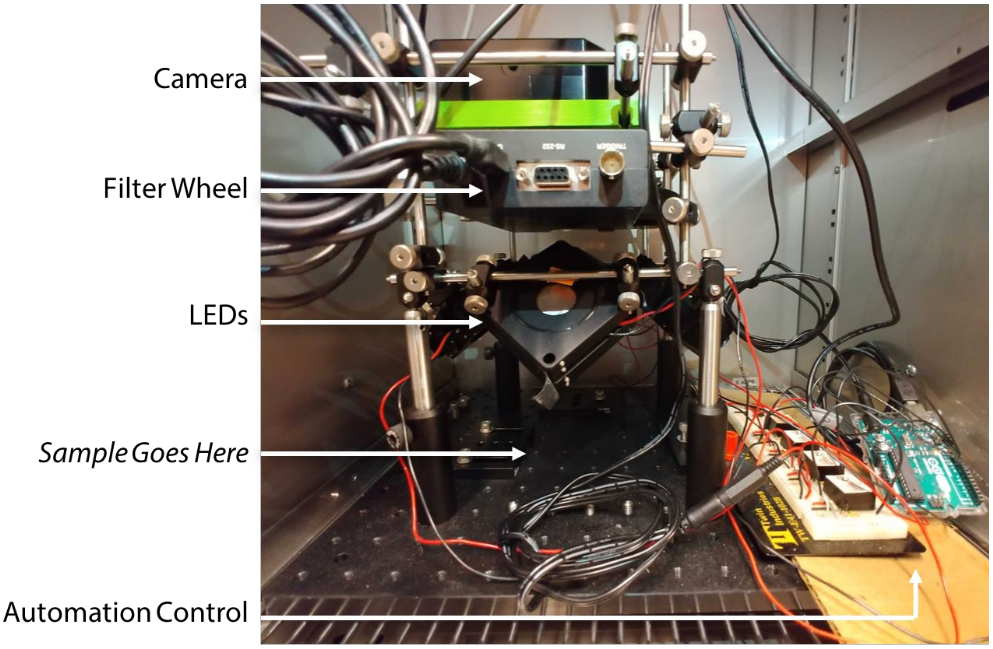

Supplemental Figure 1: Fluorescent imaging infrastructure.

This custom-built imaging device is designed to allow fluorescent imaging inside an incubator (see methods).

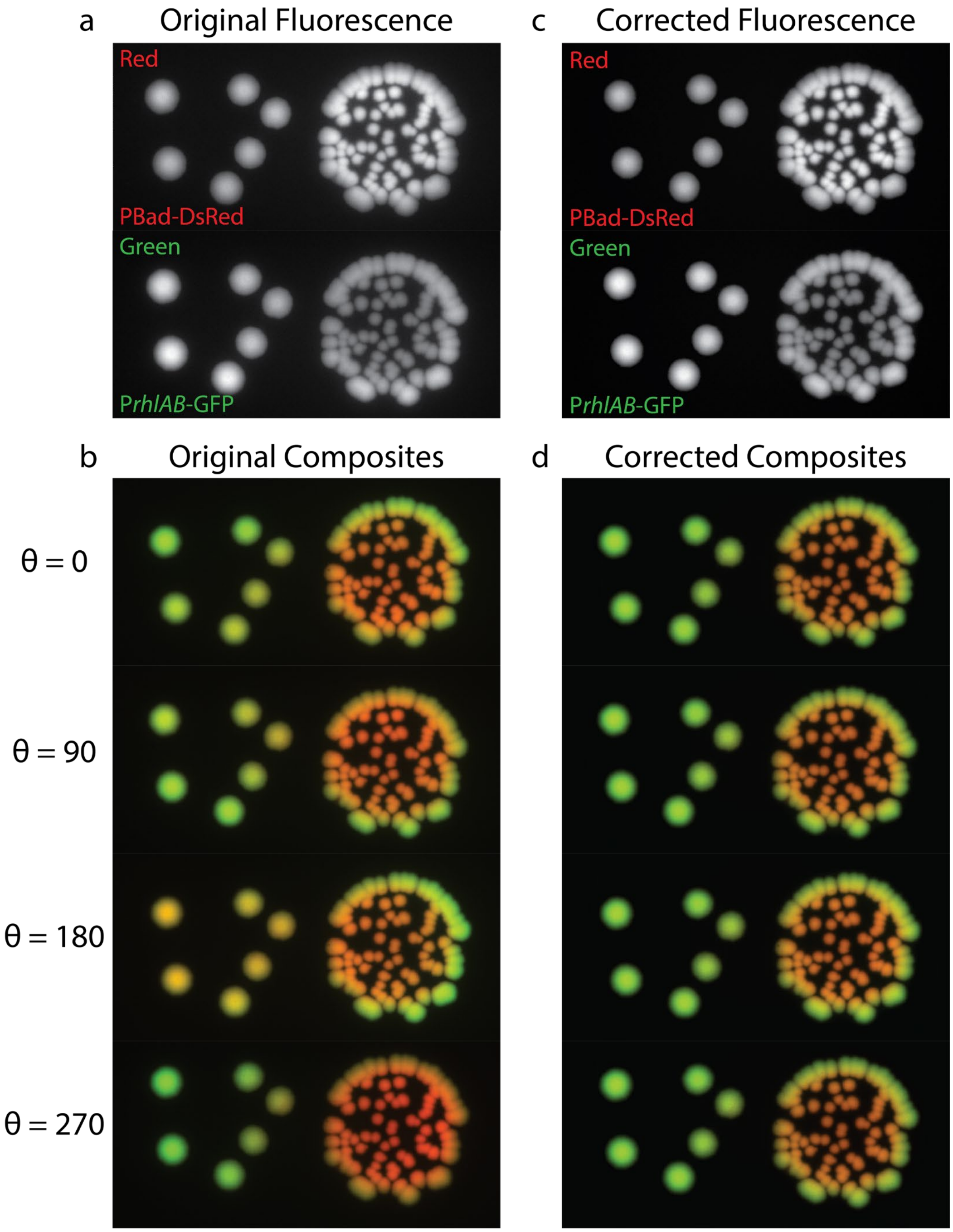

Supplemental Figure 2: Background Correction. a. 48-hour images before background correction [top] DsRed fluorescence [bottom] GFP fluorescence **b.** 48-hour RGB composite images before background correction **c.** 48-hour images after background correction [top] DsRed fluorescence [bottom] GFP fluorescence **d.** RGB composite images after background correction (see methods)

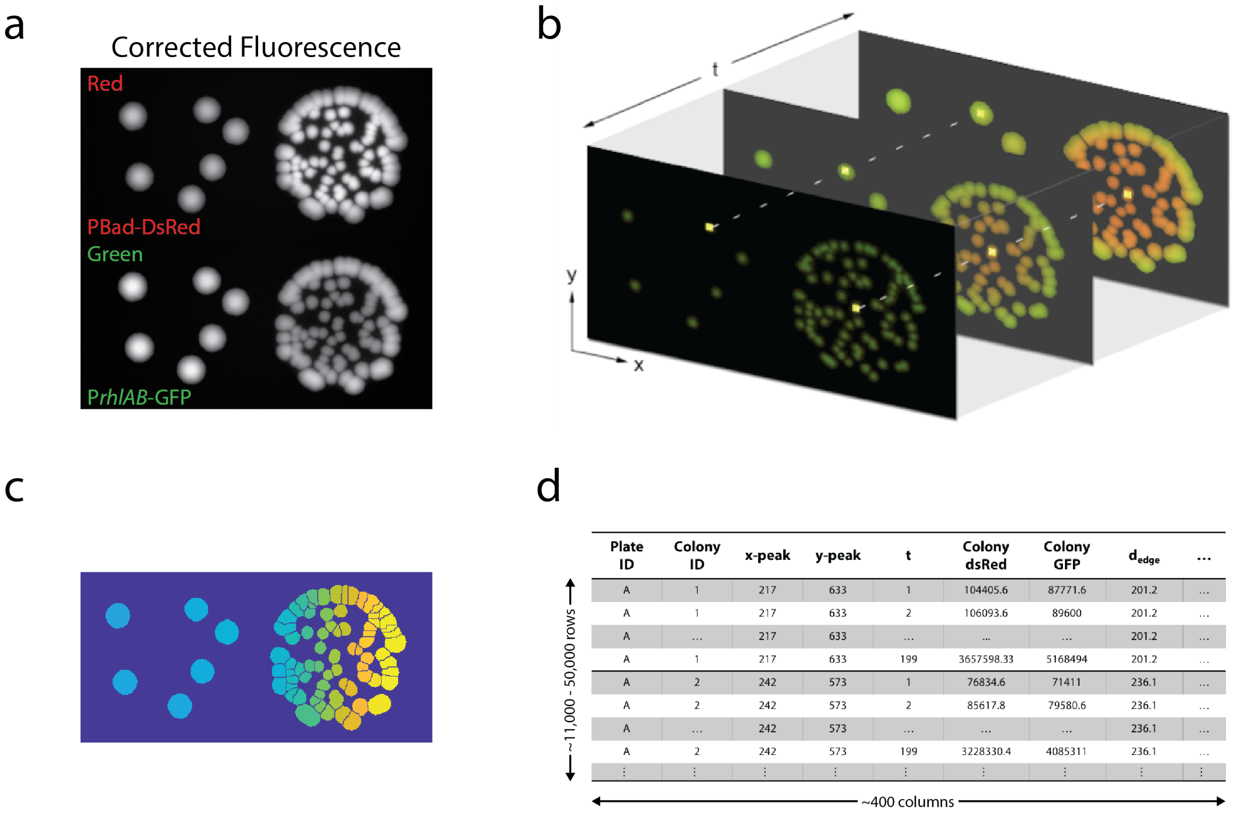

Supplemental Figure 3: Pipeline Workflow. a. CFU images after background correction **b.** Visualization of pixel-based approach for image timeseries data extraction. **c.** Pixels are collated using a custom image analysis algorithm within each colony and tracked across time. Final pixel to colony allocation map shown. **d.** Mockup of a pipeline output data frame.

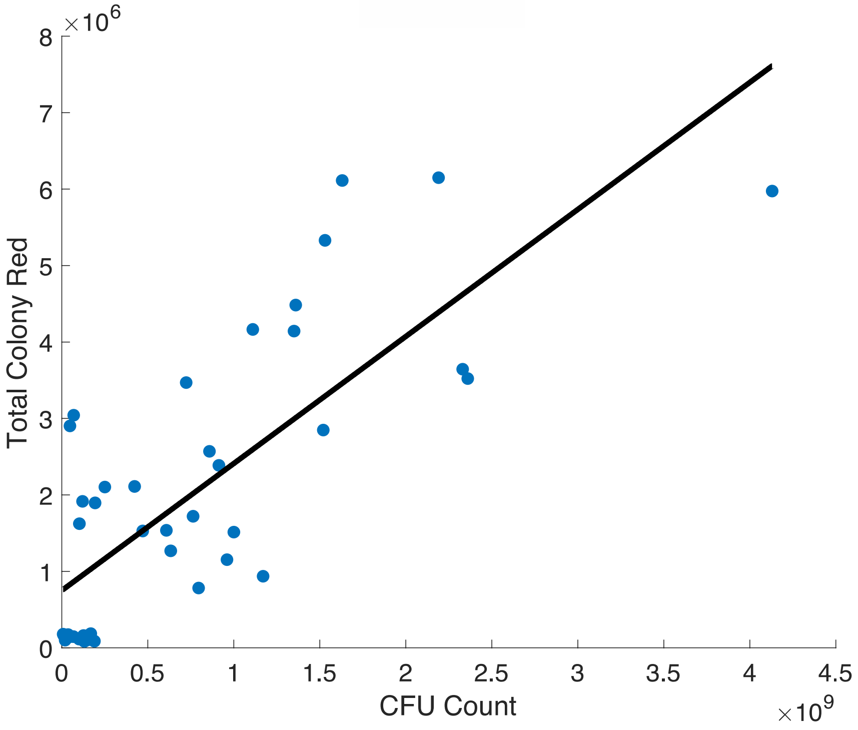

Supplemental Figure 4: DsRed as a proxy for colony biomass.

The total fluorescence for the colonies picked was compared to the colony's CFU count. Fluorescence data was collected by scanning CFU plates on a flatbed plate scanner. Colonies were picked at various growth stages and assayed for total CFU. $R^2 = 0.6006$.

Supplemental Figure 5: qPCR validation of P_{rhlAB}-GFP in liquid culture and spatially-structured systems.

[Top] WT PA14 P_{rhlAB}-GFP and P_{BAD}-DsRed cells were grown in liquid were grown in a dilution series. OD and GFP levels were measured and mRNA extracted after 24 hours. qPCR fold change data was calculated by the ΔΔCT method. Correlation coefficients were calculated on ΔCT values and GFP/OD. Coloration indicates OD at time of extraction. **a. E**xpression levels of GFP and *rhlAB* match well. $R^2 = 0.96$. **b.** Expression level of GFP correlates with GFP/OD well. $R^2 = 0.78$ **c.** Expression level of *rhlAB* level correlates with GFP/OD well. $R^2 = 0.80$.

[Bottom] PA14 Δ*lasI* Δ*rhlI* P_{rhlAB}-GFP and P_{BAD}-DsRed cells were grown on agar plates with 1μM 3-oxo-C12-D-HSL in the plate media. Each plate contained C4-HSL in a logarithmic titration from 0 to 0.5μM. DsRed and GFP levels were measured and mRNA extracted after 24 hours. Correlation coefficients were calculated on ΔCT values and GFP/DsRed. Coloration indicates the concentration of C4-HSL used. **d.** Expression levels of GFP and *rhlAB* correlate well. $R^2 = 0.93$. **e.** GFP expression level level correlates with GFP/DsRed. $R^2 = 0.94$ **f.** *rhlAB* expression level correlates with GFP/DsRed. $R^2 = 0.93$. **g.** Raw data from plates used for qPCR in d-f. Top shows image fluorescence data from the red channel, bottom shows image fluorescence data from the green channel. Plates are arranged in order of increasing C4-HSL concentration. All biomass on the plate was used for qPCR.

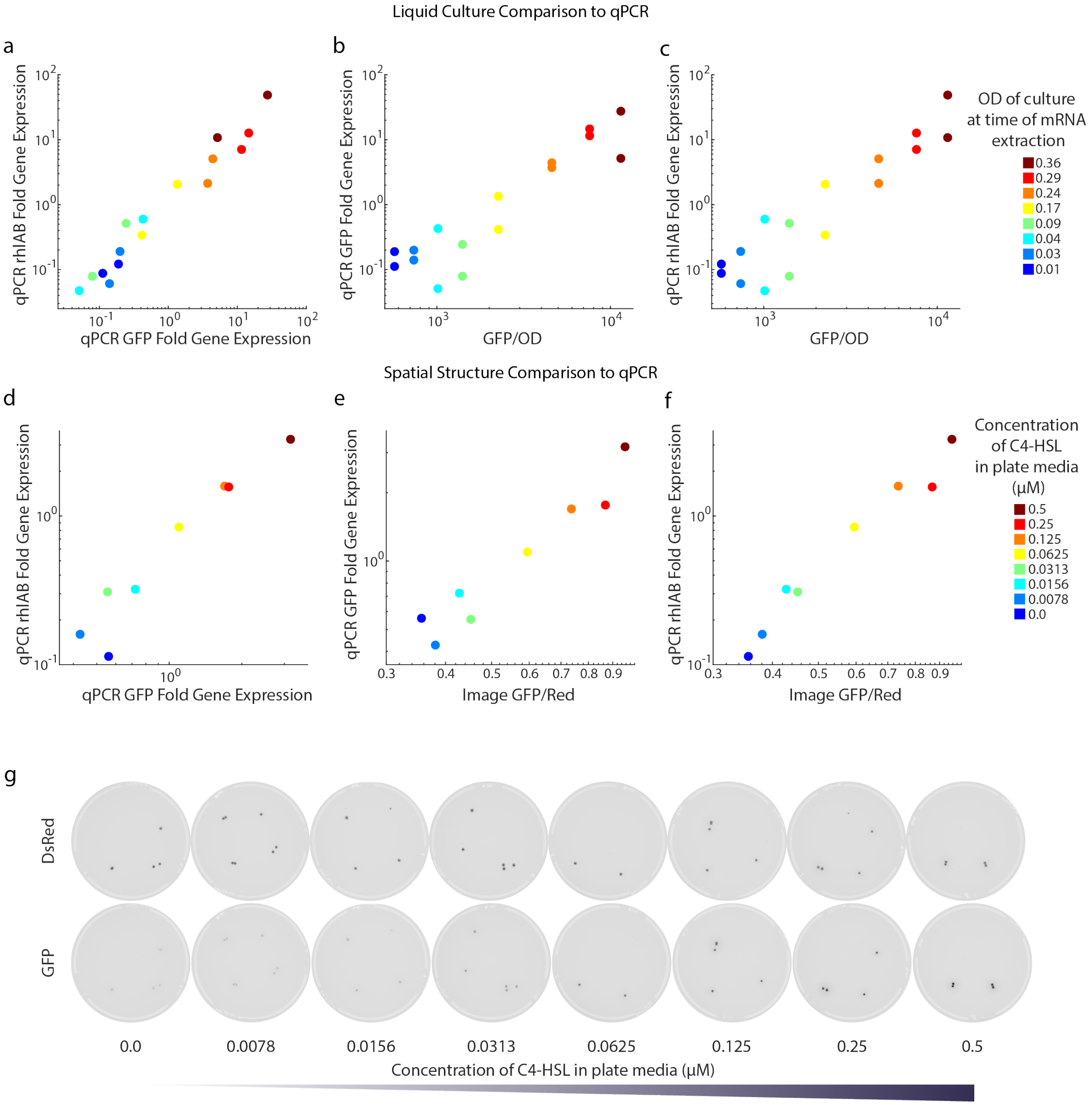
Liquid Culture Comparison to qPCR
a
b
c
qPCR rhlAB Fold Gene Expression
qPCR GFP Fold Gene Expression
GFP/OD
OD of culture at time of mRNA extraction
0.36
0.29
0.24
0.17
0.09
0.04
0.03
0.01
Spatial Structure Comparison to qPCR
d
e
f
Image GFP/Red
Concentration of C4-HSL in plate media (μM)
0.5
0.25
0.125
0.0625
0.0313
0.0156
0.0078
0.0
g
DsRed
GFP
0.0
0.0078
0.0156
0.0313
0.0625
0.125
0.25
0.5
Concentration of C4-HSL in plate media (μM)

Supplemental Figure 6: Quorum signal perturbation reveals no growth impact [Left] colonies grown without quorum signals. Data consists of 4 biological replicates including 6, 7, 39 and 39 colonies respectively. [Right] colonies grown with quorum signals in the plate media. [Top] Example data showing biomass at 48 hours. Scalebar 1 cm. Data consists of 3 biological replicates including 36, 64, and 12 colonies respectively. All statistical comparisons and resulting p-values calculated with the Mann-Whitney test. For each boxplot, the center line indicates the median. Top and bottom edges of the box indicate the 75th and 25th percentiles of the data, respectively. Whiskers indicate non-outlier extrema of the data. '+' marks indicate outliers. **a.** Final colony biomass at 48 hours. **b.** Growth rate at the time of colony appearance. **c.** Time of colony appearance.

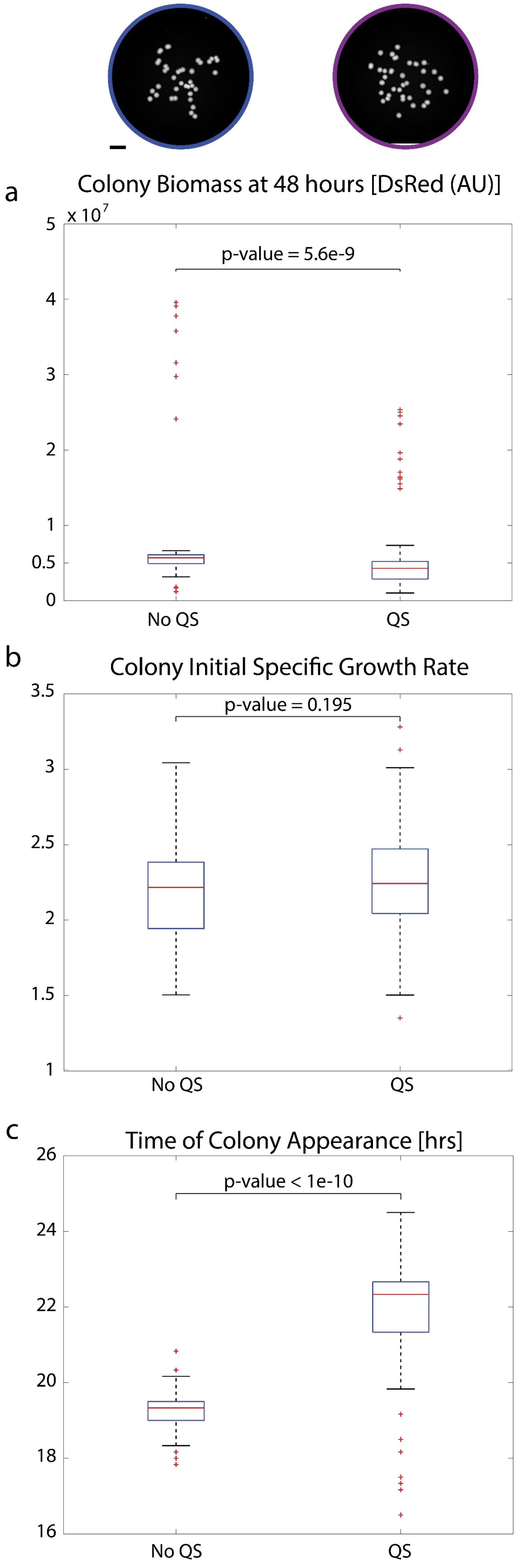

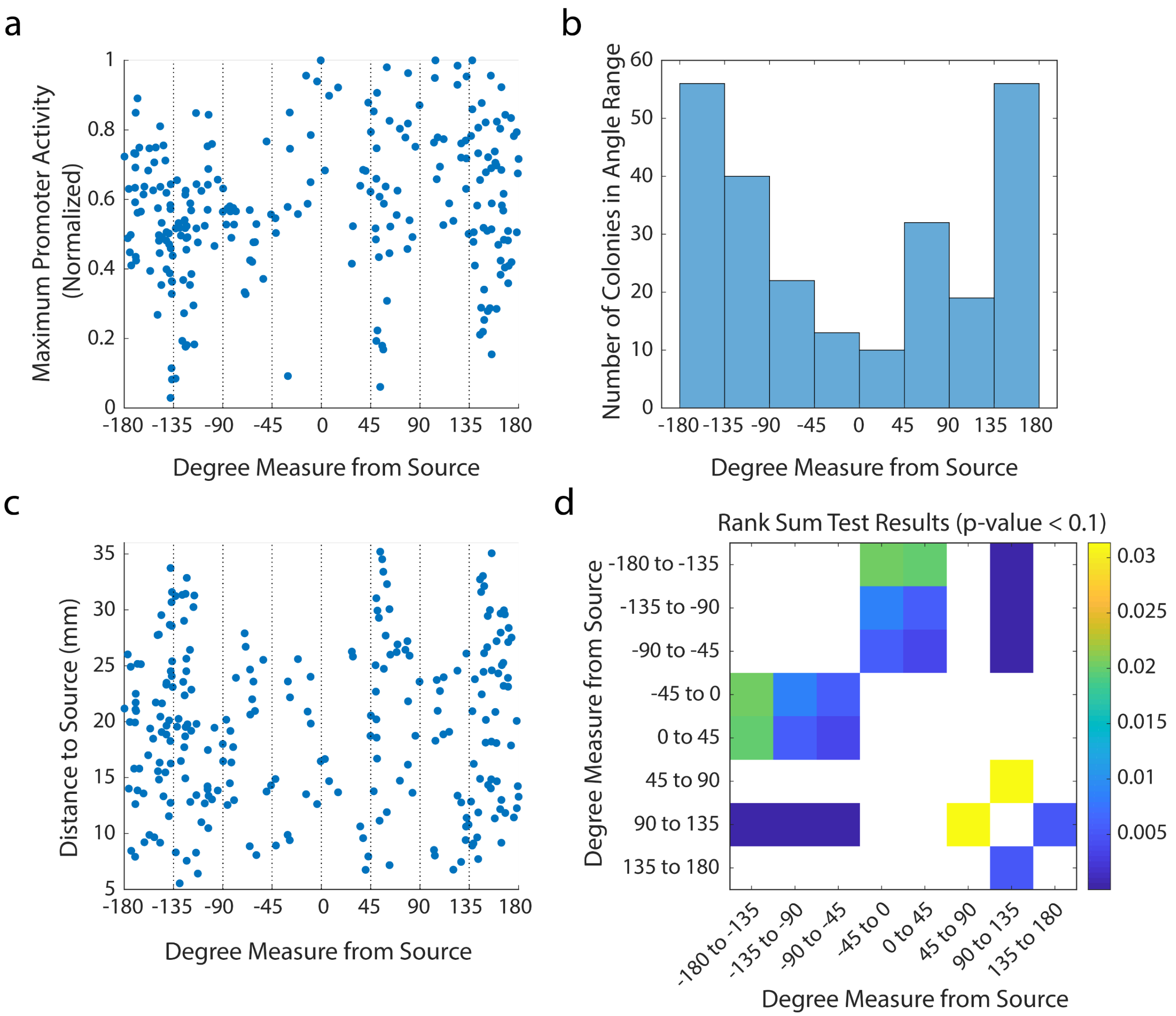

Supplemental Figure 7: Exploration of rotational distribution of colonies in Receiver datasets

a. Distribution of maximum promoter activity recorded for all colonies in Source-Receiver datasets (Figure 2) with respect to their radial location. **b.** Histogram depicting the number of colonies in each 45° increment as seen in **a**. **c.** Polar coordinate distribution of colonies as described by their distance and degree measure relative to the quorum signal source. **d.** The distribution of maximum promoter activity within each 45° increment was compared against every other increment using the two-sided rank sum test to look for any present rotational bias in these data. A systemic rotational bias would appear as an entire row and corresponding column showing a distribution that differs from all other increment distributions. All statistical comparisons performed with the Mann-Whitney test. P-values below 0.1 are shown.

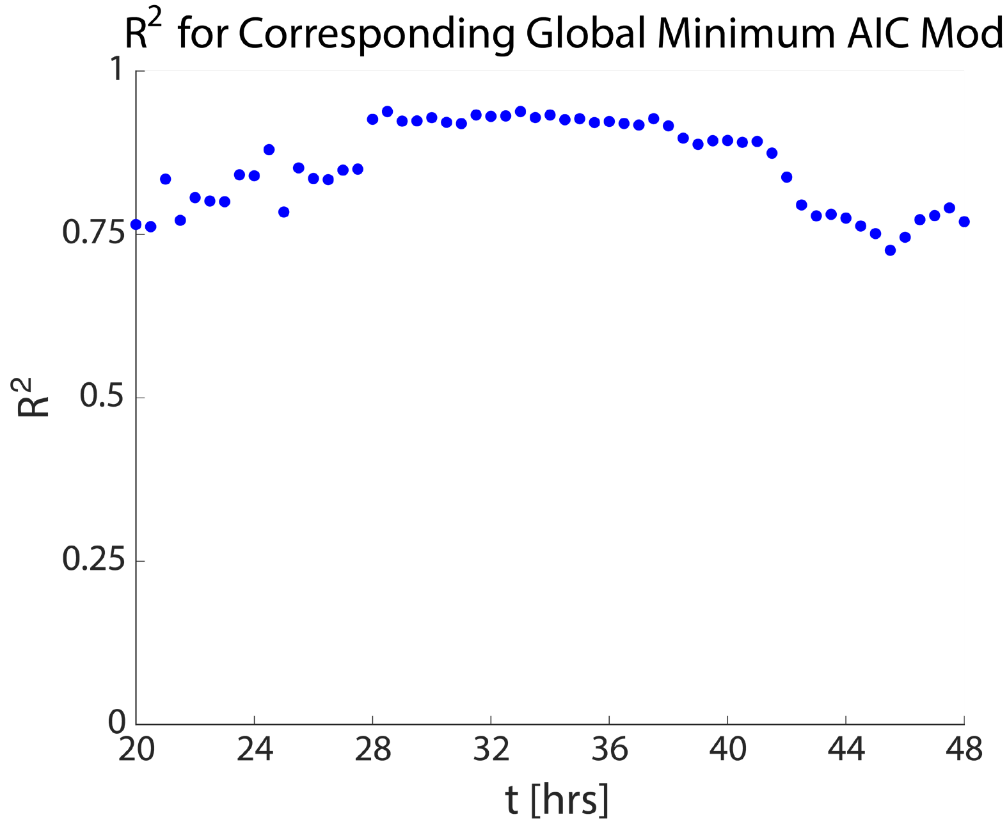

Supplemental Figure 8: Goodness of fit for models selected by AIC

Each point represents the adjusted R^2 value calculated for the corresponding model selected by global minimum AIC (Figure 3d). MATLAB calculates the adjusted R^2 as follows:

$$R^2_{adjusted} = 1 - \left(\frac{n-1}{n-p}\right)\frac{SSE}{SST} \qquad (1)$$

where n is the total number of observations, p is the number of regression coefficients (intercept inclusive), SSE is the sum of squared error and SST is the sum of squared total.

Supplemental Figure 9: Background corrected images of the swarms at various timepoints. [Left column] Raw DsRed intensity data for WT swarm at various timepoints. [Right columns] Raw GFP intensity data for WT swarm at various timepoints. Data is pseudo-colored to and scaled to the maximum intensity in that timepoint (per row). Scalebar 1 cm.

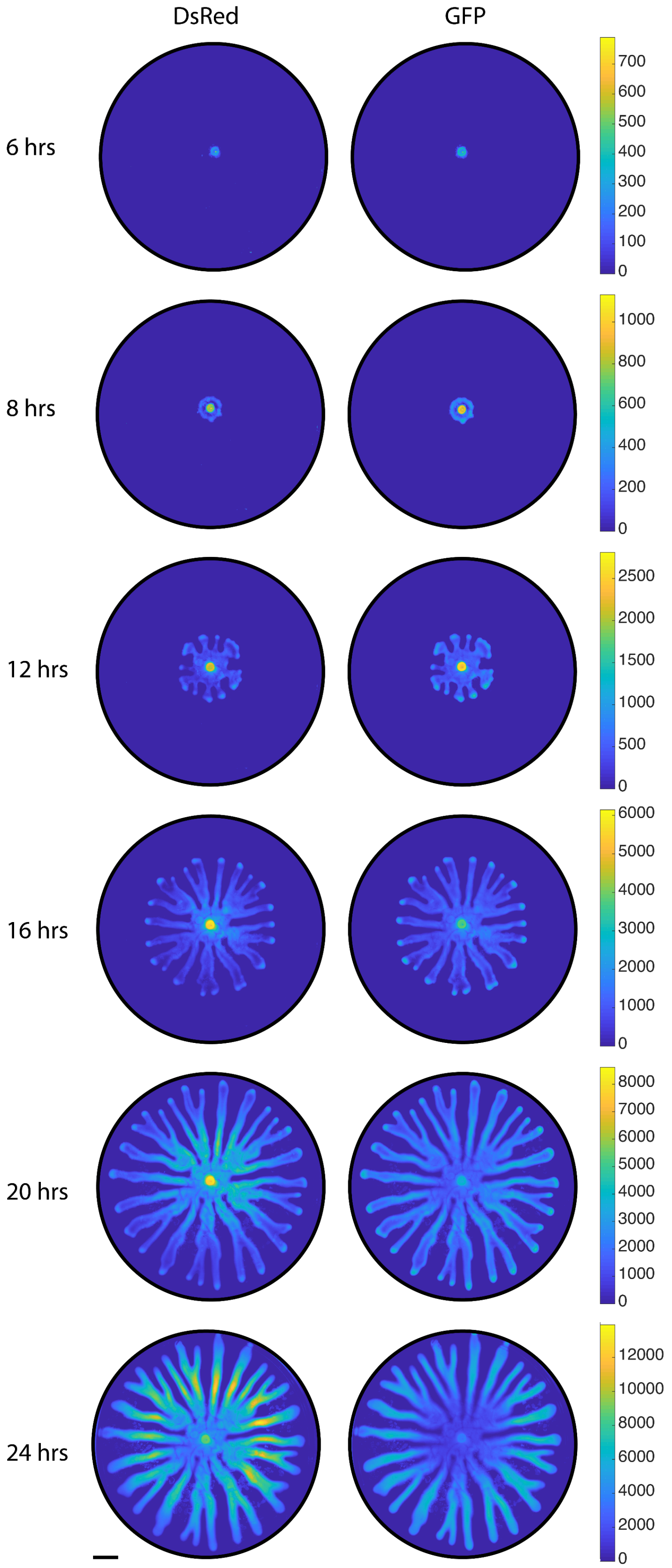

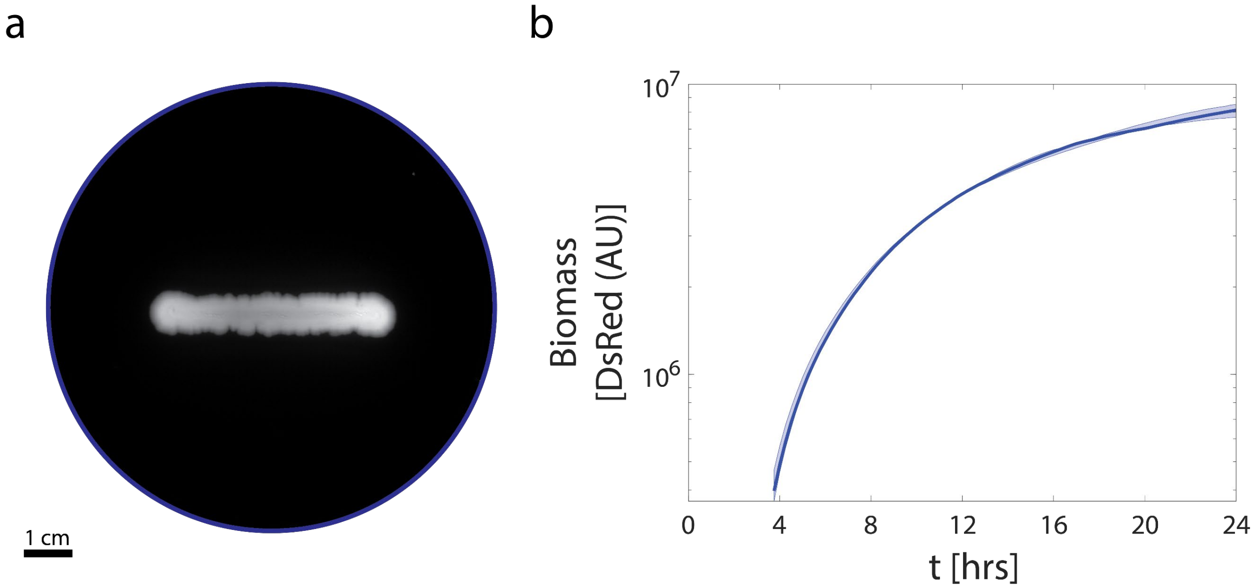

Supplemental Figure 10: Immotile tendrils show growth saturation. a. Image of biomass distribution an immotile tendril experiment at 48 hours. Cells are labeled fluorescently with DsRed, induced by the PBad promoter and corresponding L-arabinose included in the plate media. **b.** Growth in the immotile tendril over time. Shaded region describes full range of the data across three biological replicates.

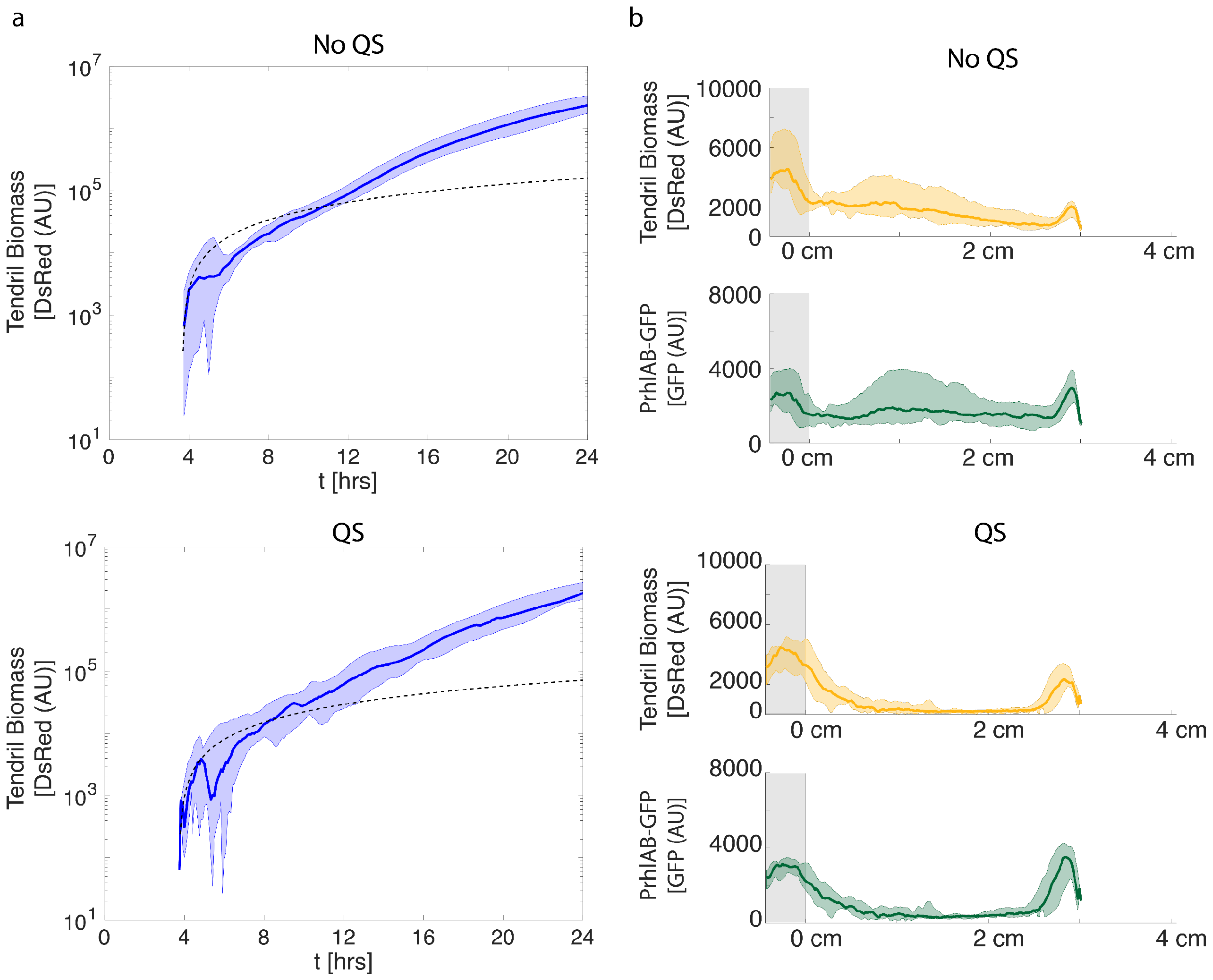

Supplemental Figure 11: Swarms perturbed with quorum signals grow similarly to non-perturbed swarms but show differences in biomass distribution.

a. Total biomass in swarming tendril over time. [Top] Swarms not given exogenous quorum signals (12 tendrils). Data repeated from Figure 4a. [Bottom] Swarms given exogenous quorum signals (9 tendrils). Dashed lines indicate linear growth dynamics. **b.** Cross-sectional distributions of biomass (DsRed) and gene expression (GFP) along 3cm length tendrils. [Top two plots] Swarms not given exogenous quorum signals (12 tendrils). Data repeated from Figure 4b. [Bottom two plots] Swarms given exogenous quorum signals (9 tendrils). Shaded regions indicate the full range of the data. Middle line indicates median data.

Supplemental Figure 12: Biomass and GFP distribution in tendrils at various lengths. [Left column] Swarm tendrils, swarms were not given quorum signals exogenously. [Right column] Swarm tendrils, swarms were given quorum signals in the plate media. Shaded regions indicate the full range of the data. Middle line indicates median data.

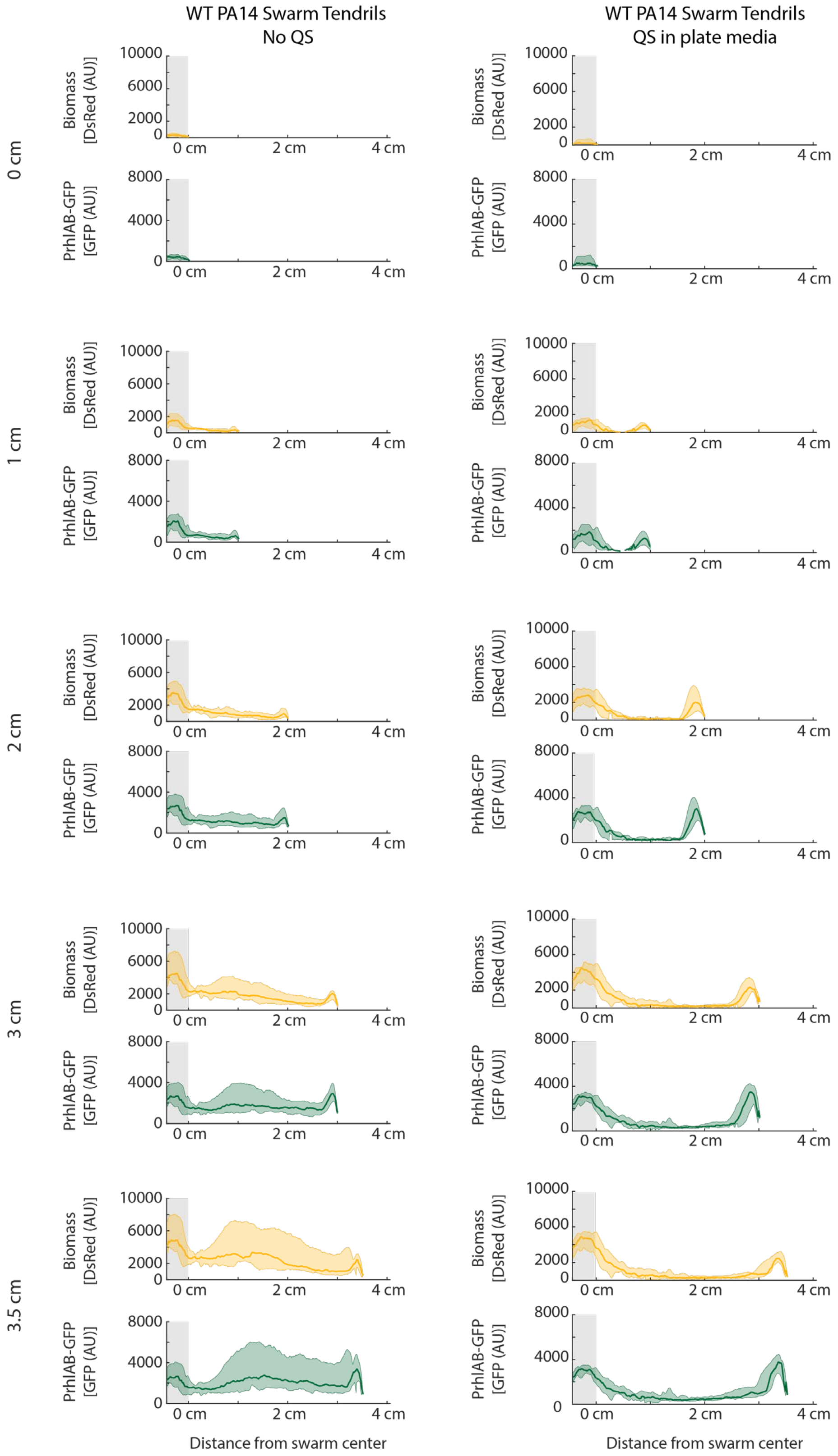

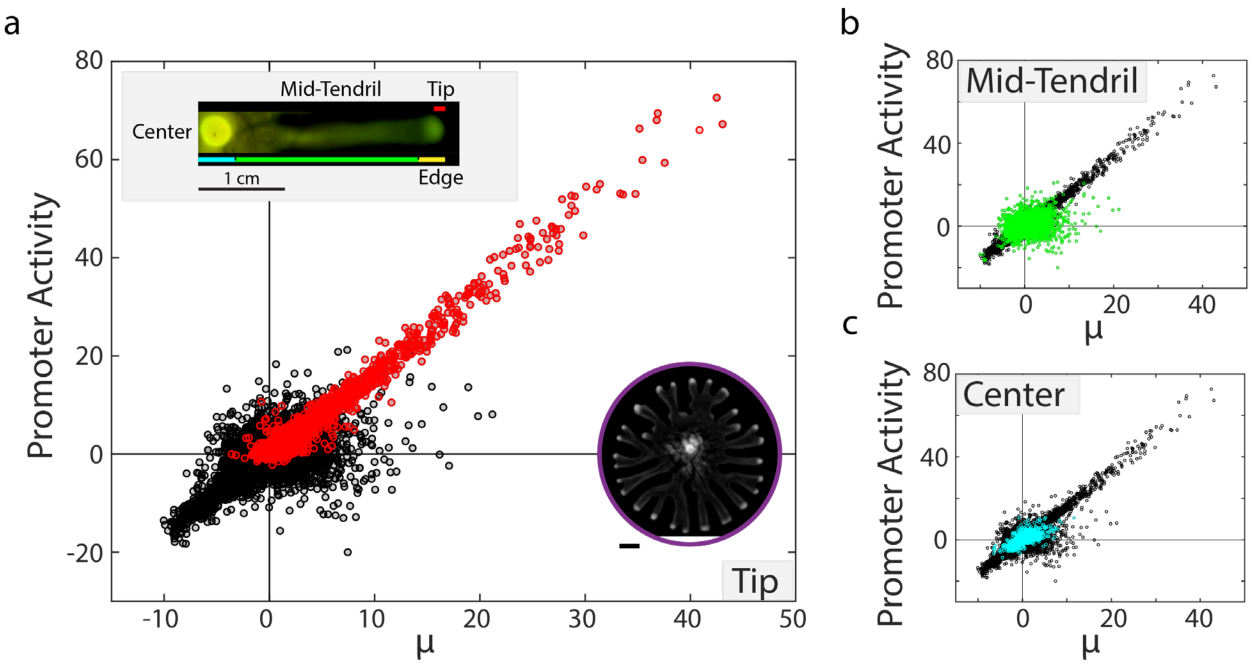

Supplemental Figure 13: Tendrils provided quorum signals show positive correlation between biomass growth rate and promoter activity.

Data shown for a representative tendril in a swarm where quorum signals were provided in the plate media. Each point represents the promoter activity and growth rate for a pixel location over a unique time interval. Color indicates the location of the pixel. **a.** Red indicates the pixel is in the 10 pixels (0.86 mm) closest to the tendril tip. [Upper inset] Definitions of tendril locations, repeated from Figure 4c. [Lower inset] Image of swarm provided quorum signals in plate media. Scale bar 1 cm. **b.** Green indicates a pixel between the tendril edge and swarm center. **c.** Cyan indicates the pixel is in the swarm center.

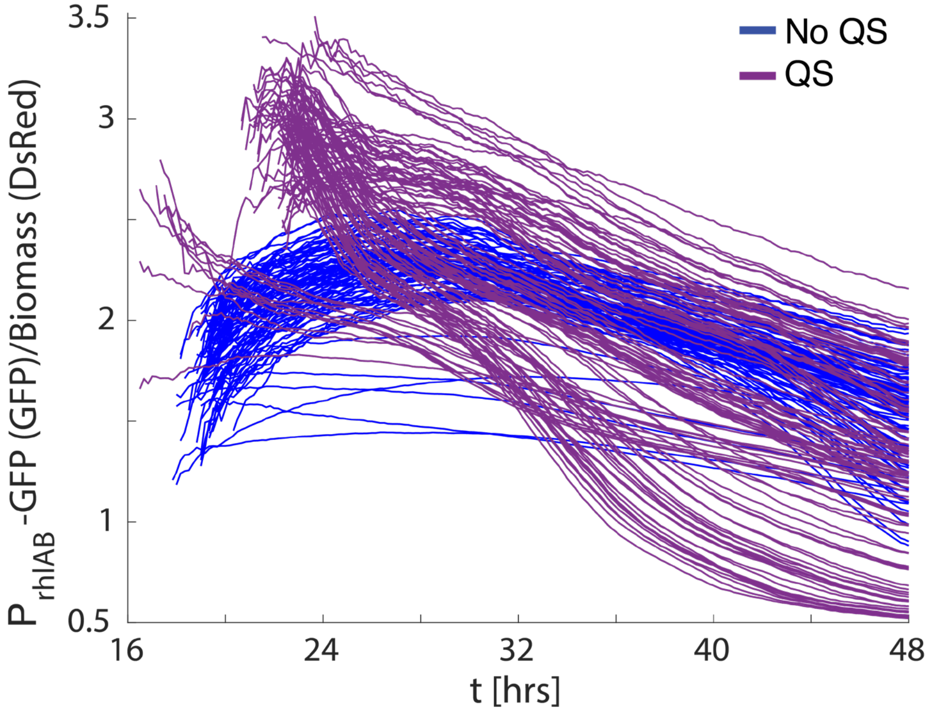

Supplemental Figure 14: Gene expression and biomass do not scale together across time. Data is the same as Figure 1d, e. Plotted is the ratio of the P_{rhlAB}-GFP to the P_{BAD}-DsRed data across time. For the CFU not provided quorum signals (blue), $R^2 = 0.307$. For the CFU provided quorum signals (purple), $R^2 = 0.542$.

Supplementary Table 1:

Competition results (WT vs Δ*rhlA* with quorum signals included in swarming plate media)

Biological Replicate	WT Initial Proportion (WT/Total)	Competition Change in Ratio (Final - Initial WT Proportion)	Final Population Size
1	0.55	0.04	6.74E+08
1	0.55	0.15	7.18E+08
1	0.55	0.03	1.72E+09
1	0.55	0.08	1.61E+09
1	0.55	0.14	7.79E+08
1	0.55	0.03	5.51E+08
1	0.55	0.06	6.39E+08
1	0.55	0.07	3.68E+08
1	0.55	0.14	5.60E+08
1	0.55	0.23	2.71E+08
1	0.55	0.10	1.23E+08
1	0.55	0.12	2.10E+08
2	0.54	0.10	7.16E+09
2	0.54	0.14	4.50E+09
2	0.54	0.09	3.71E+09
2	0.54	0.10	7.93E+09
2	0.54	0.11	8.94E+09
2	0.54	0.13	1.69E+10
2	0.54	0.11	1.64E+10
2	0.54	0.09	3.52E+09
2	0.54	0.12	6.62E+09
2	0.54	0.11	8.56E+09
2	0.54	0.14	7.11E+09
2	0.54	0.10	1.31E+10
3	0.49	0.05	2.43E+09
3	0.49	0.14	4.87E+09
3	0.49	0.09	1.56E+10
3	0.49	0.14	2.94E+09
3	0.49	0.12	5.83E+09
3	0.49	0.08	7.26E+09
3	0.49	0.01	8.59E+09
3	0.49	0.12	7.28E+09
3	0.49	0.10	1.27E+10
3	0.49	0.12	1.06E+10
3	0.49	0.11	4.59E+09

Supplementary Table 2:

Media Recipe

**Agar Plate Media (makes 12 plates)**
100 mL Agar*
98.5 mL H_2O
50 mL 5x Minimal Salts**
250 µL 1M $MgSO_4$
25 µL 1M $CaCl_2$
6.25 mL 200g/L Casamino Acids
1.8mL 40% w/v L-arabinose

*Replaced with water in liquid culture assays.
**Mixture was autoclaved and contained 64g $Na_2HPO_47H_2O$, 15g KH_2PO_4,2.5g NaCl with water added to make 1L of solution.

Supplementary Table 3:

qPCR Primers (2)

Gene of Interest	Forward Primer	Reverse Primer
proC (Housekeeping gene used for reference)	GTG GTC CTG TCG GTC AAG	GAT GGA GAC GAT CAG TTG CTC
rhlA	GGC GCG AAA GTC TGT TGG T	CCA ACG CGC TCG ACA TG
GFP	GAT GGT GAT GTT AAT GGG CAC	GGG TAA GTT TTC CGT ATG TTG C

Supplementary References

1. Jonathan C. Lansey (2021). Plot and compare histograms; pretty by default (https://www.mathworks.com/matlabcentral/fileexchange/27388-plot-and-compare-histograms-pretty-by-default), MATLAB Central File Exchange. Retrieved Feb 9, 2021.
2. Boyle, K.E., Monaco, H.T., Deforet, M., Yan, J., Wang, Z., Rhee, K., and Xavier, J.B. (2017). Metabolism and the evolution of social behavior. Mol. Biol. Evol. *34*, 2367–2379.
