# Supplementary material for: Spatial-temporal dynamics of a microbial cooperative behavior resistant to cheating
Source: Nat Commun. 2022 Feb 7;13:721. doi: 10.1038/s41467-022-28321-9 (PMC8821651; doi:10.1038/s41467-022-28321-9)
Supplement: Supplementary file 3 — Reporting summary [file 41467_2022_28321_MOESM3_ESM.pdf]

nature research

Corresponding author(s): Joao B. Xavier and Hilary Monaco

Last updated by author(s): Nov 21, 2021

Reporting Summary

Nature Research wishes to improve the reproducibility of the work that we publish. This form provides structure for consistency and transparency in reporting. For further information on Nature Research policies, see our Editorial Policies and the Editorial Policy Checklist.

Statistics

For all statistical analyses, confirm that the following items are present in the figure legend, table legend, main text, or Methods section.

n/a	Confirmed	
☐	☒	The exact sample size (n) for each experimental group/condition, given as a discrete number and unit of measurement
☐	☒	A statement on whether measurements were taken from distinct samples or whether the same sample was measured repeatedly
☐	☒	The statistical test(s) used AND whether they are one- or two-sided<br>*Only common tests should be described solely by name; describe more complex techniques in the Methods section.*
☐	☒	A description of all covariates tested
☐	☒	A description of any assumptions or corrections, such as tests of normality and adjustment for multiple comparisons
☐	☒	A full description of the statistical parameters including central tendency (e.g. means) or other basic estimates (e.g. regression coefficient) AND variation (e.g. standard deviation) or associated estimates of uncertainty (e.g. confidence intervals)
☐	☒	For null hypothesis testing, the test statistic (e.g. F, t, r) with confidence intervals, effect sizes, degrees of freedom and P value noted<br>*Give P values as exact values whenever suitable.*
☐	☒	For Bayesian analysis, information on the choice of priors and Markov chain Monte Carlo settings
☐	☒	For hierarchical and complex designs, identification of the appropriate level for tests and full reporting of outcomes
☒	☐	Estimates of effect sizes (e.g. Cohen's d, Pearson's r), indicating how they were calculated

Our web collection on statistics for biologists contains articles on many of the points above.

Software and code

Policy information about availability of computer code

Data collection	Liquid culture data was collected by a TECAN M1000. Image data was collected by an Atik VS14 fluorescent camera through the Thorlabs filter wheel FW102C. Timeseries images were collected through a custom built control system using the Arduino Uno R3. The code for the control system was written in MATLAB and is available upon request.
Data analysis	All data was processed using MATLAB 2018a. Image processing software is available in the GitHub respository at https://github.com/htlm/Git-ImageAnalysis.

For manuscripts utilizing custom algorithms or software that are central to the research but not yet described in published literature, software must be made available to editors and reviewers. We strongly encourage code deposition in a community repository (e.g. GitHub). See the Nature Research guidelines for submitting code & software for further information.

Data

Policy information about availability of data

All manuscripts must include a data availability statement. This statement should provide the following information, where applicable:

- Accession codes, unique identifiers, or web links for publicly available datasets
- A list of figures that have associated raw data
- A description of any restrictions on data availability

The data generated in this study are available in the Supplementary Information and the Source Data file. The raw data can be found at https://figshare.com/projects/Spatial-temporal_microbial_cooperation/124954.

Field-specific reporting

Please select the one below that is the best fit for your research. If you are not sure, read the appropriate sections before making your selection.

☐ Life sciences ☐ Behavioural & social sciences ☒ Ecological, evolutionary & environmental sciences

For a reference copy of the document with all sections, see nature.com/documents/nr-reporting-summary-flat.pdf

Ecological, evolutionary & environmental sciences study design

All studies must disclose on these points even when the disclosure is negative.

Study description

This study quantifies and compares the growth and gene expression (P_{rhlAB}) of Pseudomonas aeruginosa in liquid and spatially-structured environments including colony forming units (CFUs) and swarms. We also examine changes in P_{rhlAB} activity in response to an exogenous quorum signal perturbation (QS). We call in these environmental conditions without QS, "No QS," and with QS, "QS."

We find patterns in P_{rhlAB} activity in CFU and swarms that differ from previously established patterns from liquid data.

Figure 1
a. n/a
b. No QS n = 36, QS n = 36, 48 hour timeseries with measurements taken every 10 minutes, 3 biological replicates including both experimental conditions. See methods for details on growth rate and promoter activity calculations.
c. No QS n = 91, QS n = 112, 48 hour timeseries with measurements taken every 10 minutes, 4 No QS and 3 QS replicate days. See methods for details on growth rate and promoter activity calculations.
d. Same as 1b
e. Same as 1c

Figure 2
a. n/a
b. n/a
c-d. n = 248 across 3 separate plates each performed on a different day

Figure 3
a-b. n/a
c-f. n = 261 taken over 3 independent days

Figure 4
a. 4 biological replicate swarms, 3 swarming tendrils from each swarm. 24 hour timeseries with measurements taken every 5 minutes
b. Same as 4a
c. Representative swarming tendril. 24 hour timeseries with measurements taken every 5 minutes. See methods for details on growth rate and promoter activity calculations

Figure 5
a. No QS data same as 4a,b. QS data contains 3 biological replicates, 3 swarming tendrils taken from each replicate.
b. No QS: 8 biological replicates with 80 total technical replicates. QS: 6 biological replicates with 54 total technical replicates.
c. Data calculated from same samples used in 5a
d. Competition assays were performed in 3 biological replicates, each with multiple technical replicates. 35 replicate competitions.

Research sample

Pseudomonas aeruginosa is an opportunistic human pathogen capable of complex multicellular social behaviors in well-mixed (liquid culture), sessile (biofilm) and motile (swarming) lifestyles. It is a primary model system for the study of microbial social behavior. This is why it was chosen for this study on social behaviors in spatially-structured systems.

Samples are populations of cells in the form of a liquid culture (150 microliter volume containing cells and growth media), a population resulting from a colony forming unit (CFU), or a swarm (see Xavier et al, 2011 for swarming protocol and media compositions). These populations are meant to capture similar cell growth dynamics across well-mixed (liquid) and spatially-structured (cCFU and swarming) systems to allow comparison of social behaviors across environments.

All samples are Pseudomonas aeruginosa. We use three different strains in this work:
- WT PA14 strain,
- A double deletion mutant (Delta rhlI Delta lasI) made from PA14 (Figure 2c, d),
- A deletion mutant (Delta rhlA) made from PA14 (competition assays, Figure 5d).

Sampling strategy

Previously published work informed our sample sizes for liquid culture and swarming competition assays (Xavier et al, 2011, de Vargas Roditi et al, 2013, and Boyle et al, 2015).

We aimed to sample the underlying distribution of growth curves and P_rhlAB_{GFP} dynamics through the observation of many colonies grown from CFU (cCFUs). We minimized variability by plating the cells while in exponential phase. Our initial investigation of

cCFU growth curves showed consistency across plates containing similar numbers of colonies in similar configurations. Given the consistency we observed, the final datasets for this study came from three (or four where indicated) CFU plates taken on separate days. The number of cCFUs we extracted from these data was at least triple the number of replicates used in our liquid culture datasets from this work and previous published work (Boyle et al, 2015, Xavier et al, 2011). We found that with this sample size, we were able to both see consistency in our data across experimental days and observe differences under experimental perturbation.

Early investigation of time to tendril formation (data not shown) revealed considerable variation in this assay. Analyses of these data informed the number of swarms analyzed for each condition.

Data collection

Liquid culture data was collected by Hilary Monaco and Kevin S. Liu via a TECAN M1000. The output data comes as an excel spreadsheet.

cCFU image timeseries, swarming timeseries assays, time to tendril formation assays and competition assays were collected by Hilary Monaco, Kevin S. Liu, Tiago Sereno and Caleb C. Reagor.
- cCFU and swarming timeseries were collected as TIFF images.
- Time to Tendril Formation data was collected in excel spreadsheets.
- Competition assay data was collected as TIFF images with data extraction performed using FIJI and was stored in excel spreadsheets.

qPCR data was collected by Hilary Monaco and Yanyan Chen using an Applied Biosystems QuantStudio 6 Flex. The output data comes as an excel spreadsheet.

Timing and spatial scale

Data were collected between January 2017- December 2019. Liquid data was collected from replicates in 150 microliter volume in 96 well plates. Spatially-structured data from CFUs or swarms were collected in 9 cm diameter petri dishes. qPCR data were collected in 2021 due to a reviewer request.

Data exclusions

One dataset was excluded due to contamination found on the petri dish. It was shown mid-study that the quorum signals used in the plate media started to degrade after one week. One dataset collected one week after the plates media was made was retroactively excluded. Three datasets were excluded due to saturation in the images due to incorrect settings used with the incubated fluorescent imager.

Reproducibility

Each experiment included in this study was repeated on three separate days. Any exceptions, as noted in the above Study Description, were performed on four separate days. Aside from aforementioned exclusions, all attempts at replication were successful.

Randomization

No randomization was relevant to this study as there were no experiments in which cell populations were assigned to treatment groups.

Blinding

Data in the "time to tendril formation" assay was collected by at least two researchers and averaged. To prevent bias in the data collection, at least one of those researchers (at least two in the case of datasets analyzed by three researchers) was blind to the randomized incubator configuration. Fluctuations in the availability of the research team made further blinding impractical.
All other data collection was performed through automated instruments without scientist involvement.

Did the study involve field work? ☐ Yes ☒ No

Reporting for specific materials, systems and methods

We require information from authors about some types of materials, experimental systems and methods used in many studies. Here, indicate whether each material, system or method listed is relevant to your study. If you are not sure if a list item applies to your research, read the appropriate section before selecting a response.

Materials & experimental systems

n/a	Involved in the study	
☒	☐	Antibodies
☒	☐	Eukaryotic cell lines
☒	☐	Palaeontology and archaeology
☒	☐	Animals and other organisms
☒	☐	Human research participants
☒	☐	Clinical data
☒	☐	Dual use research of concern

Methods

n/a	Involved in the study	
☒	☐	ChIP-seq
☒	☐	Flow cytometry
☒	☐	MRI-based neuroimaging
