# Supplementary figures and images for: Spatial-temporal dynamics of a microbial cooperative behavior resistant to cheating
Source: Nat Commun. 2022 Feb 7;13:721. doi: 10.1038/s41467-022-28321-9 (PMC8821651; doi:10.1038/s41467-022-28321-9)

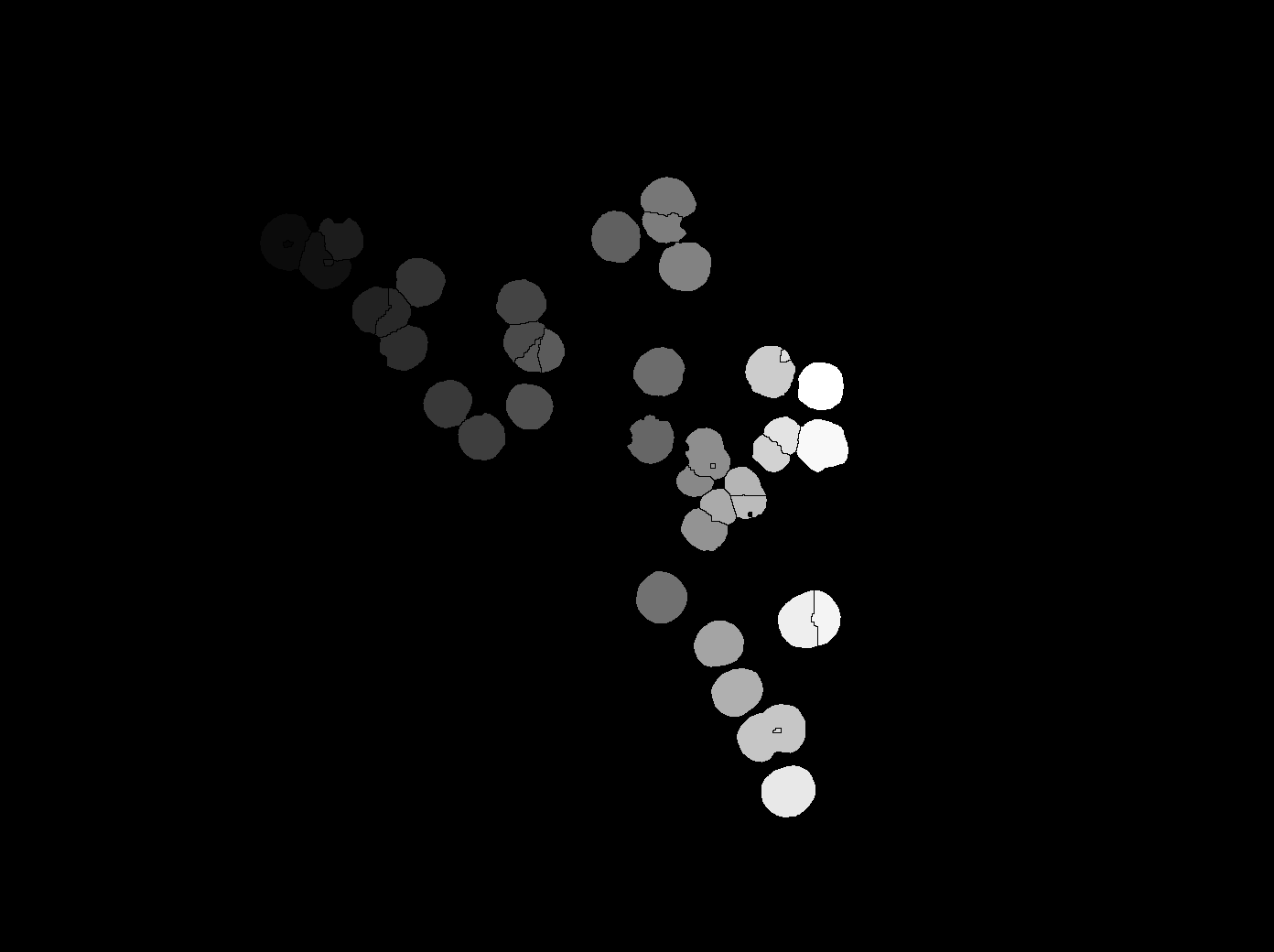

Supplement: Supplementary file 4 — Source Data [file 41467_2022_28321_MOESM4_ESM.zip › Figure_Source_Data_Final/Final_Fig2_Colony_Map.tif]

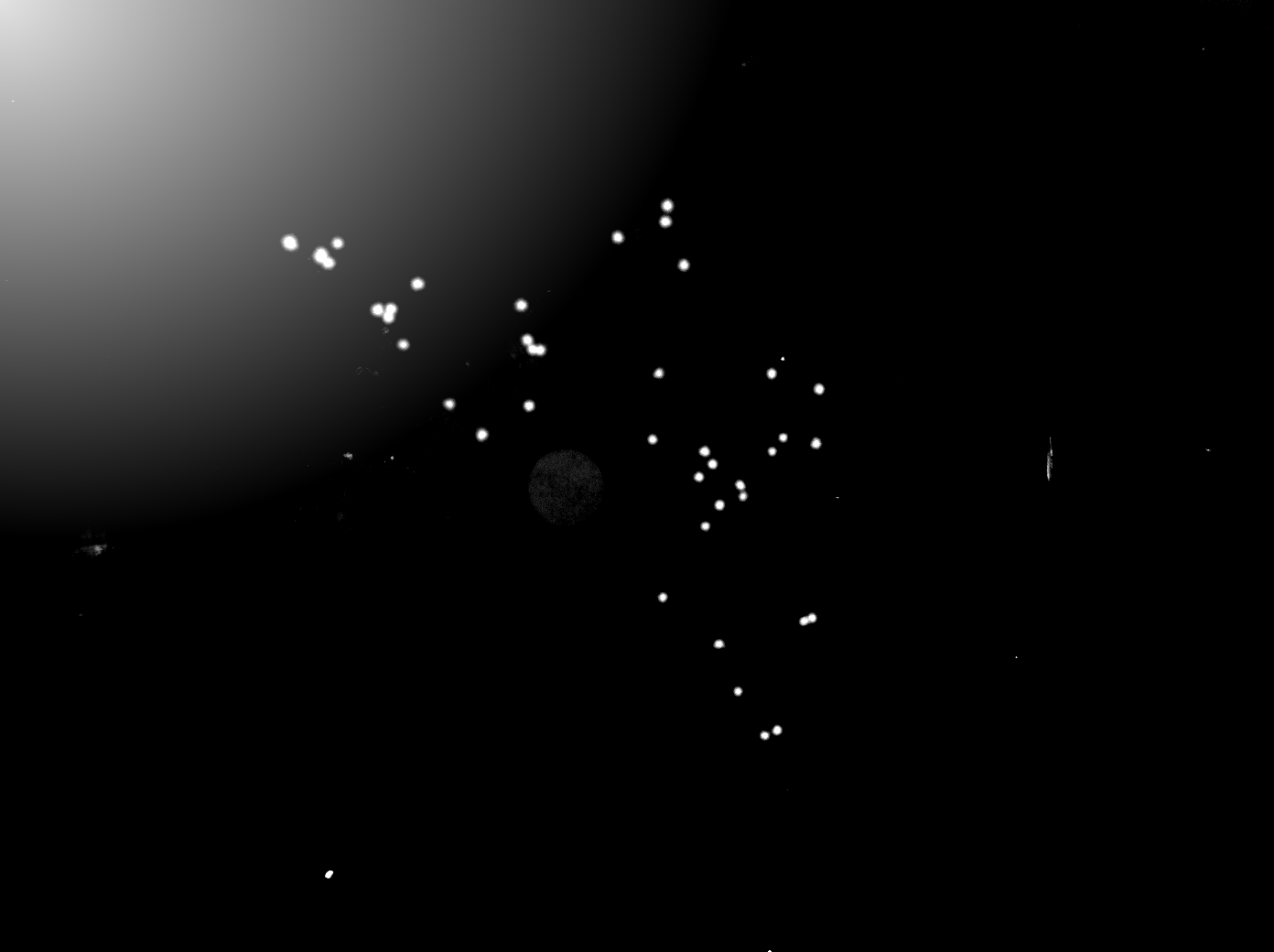

Supplement: Supplementary file 4 — Source Data [file 41467_2022_28321_MOESM4_ESM.zip › Figure_Source_Data_Final/Final_Fig2_Gradient_Image_Biomass.tif]

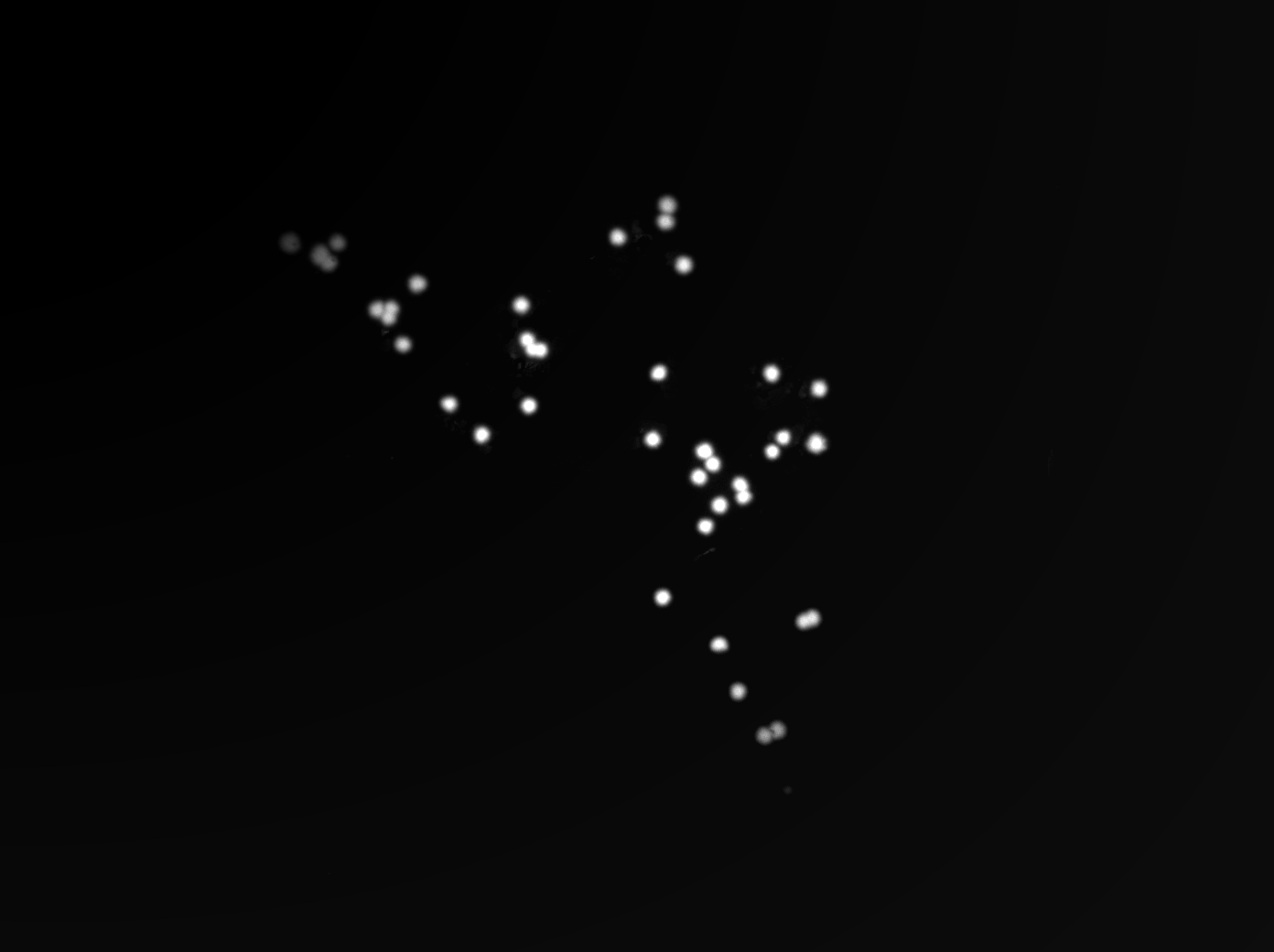

Supplement: Supplementary file 4 — Source Data [file 41467_2022_28321_MOESM4_ESM.zip › Figure_Source_Data_Final/Final_Fig2_Gradient_Image_GFP.tif]
